# Accelerated Reliability Growth Test for Magnetic Resonance Imaging System Using Time-of-Flight Three-Dimensional Pulse Sequence

**DOI:** 10.3390/diagnostics9040164

**Published:** 2019-10-25

**Authors:** Pradeep Kumar Anand, Dong Ryeol Shin, Navrati Saxena, Mudasar Latif Memon

**Affiliations:** 1 College of Information and Communication Engineering, Sungkyunkwan University, Suwon 16419, Korea; pradeep@skku.edu; 2 College of Software, Sungkyunkwan University, Suwon 16419, Korea; navrati@skku.edu; 3 IBA Community College Naushahro Feroze, Sukkur IBA University, Sindh 65200, Pakistan; mudasarlatif.ccnf@iba-suk.edu.pk

**Keywords:** system reliability test, growth test, Crow AMSAA, magnetic resonance imaging, MRI, MRI usage scenario, accelerated test

## Abstract

A magnetic resonance imaging (MRI) system is a complex, high cost, and long-life product. It is a widely known fact that performing a system reliability test of a MRI system during the development phase is a challenging task. The major challenges include sample size, high test cost, and long test duration. This paper introduces a novel approach to perform a MRI system reliability test in a reasonably acceptable time with one sample size. Our approach is based on an accelerated reliability growth test, which consists of test cycle made of a very high-energy time-of-flight three-dimensional (TOF3D) pulse sequence representing an actual hospital usage scenario. First, we construct a nominal day usage scenario based on actual data collected from an MRI system used inside the hospital. Then, we calculate the life-time stress based on a usage scenario. Finally, we develop an accelerated reliability growth test cycle based on a TOF3D pulse sequence that exerts highest vibration energy on the gradient coil and MRI system. We use a vibration energy model to map the life-time stress and reduce the test duration from 537 to 55 days. We use a Crow AMSAA plot to demonstrate that system design reaches its useful life after crossing the infant mortality phase.

## 1. Introduction

A system reliability test of a high-cost and long-life repairable product during the development phase and prior to product launch is a big challenge [1,2,3,4]. A magnetic resonance imaging (MRI) system costs approximately one million USD and has more than 10 years of product life [1]. Several attempts are made by top MRI companies in the world to perform their systems’ reliability tests during the product development phase. However, these companies face several challenges in terms of test cost, test time, and sample size [1,2,3]. Higher system reliability of a product results in higher availability and less maintainability [3]. Hence, higher system reliability results in less cost to the customer [3]. As on today, MRI companies perform parts and software reliability tests to achieve product reliability. Parts and software reliability tests are relatively well proven concepts, easy to perform, take less test time and cost less compared to a system reliability test [4]. However, they lack the capacity to identify unknown and hidden failures especially due to complex interaction between hardware–hardware, hardware–software, and software–software in a product. These unknown and hidden failures result in product defects and hence poor reliability [4]. To perform a MRI system reliability test during product development phase, we learned several system reliability test techniques from other industries. These techniques are reliability growth test [4,5,6,7], reliability demonstration test [2], Crow Army Material Systems Analysis Activity (AMSAA) test [8,9,10,11], life test [12,13], accelerated life test [14], and burn in test [15] etc. One of the challenges to perform the MRI system reliability test is sample size due to very high sample cost. To resolve the sample size issue, we research further and find a solution to perform the reliability growth and demonstration test on one sample size for a high-cost and long-life product [2].

We propose a novel approach to perform a MRI system accelerated reliability growth test based on a hospital usage scenario on one sample size in a reasonably acceptable time. First, we develop a MRI usage scenario based on actual uses inside the hospital. Based on the usage scenario, we identify the stress conditions and parameters. MRI systems stress heavily while running pulse sequences during a patient scan. After analyzing further, we discover that during pulse sequences, MRI gradient coil vibration energy represents best the stress parameters. Once stress conditions and parameters are identified, we estimate the life-time stress for a MRI system as per hospital usage scenario. Based on life-time stress, we calculate the time to complete the MRI system reliability growth test. The test duration was 537 days, which was extremely high and unacceptable to any product development company. To resolve this issue, we identify a time-of-flight three-dimensional (TOF3D) pulse sequence stress. Hence, using TOF3D pulse sequence, we developed a test cycle to accelerate the reliability growth test. The test time reduced from 537 day to 55 days. The approach was successfully tested on a MRI system. During the test, several hidden and unknown failures were discovered. Test results were further analyzed using the Crow AMSAA concept by plotting failures with respect to test time. A Crow AMSAA plot shows graphically that system reliability and design maturity were achieved, which also helped to terminate the test.

The rest of the paper is organized as follows. Section 2 presents a brief overview of a MRI system and different types of system level reliability tests as part of related work. Our proposed method for developing nominal usage scenario of a MRI system based on hospital field data and workflow is described in Section 3. This section also presents the current challenges to perform a MRI system reliability growth test in addition to identifying the system stress condition and stress parameters. In Section 3, test sequence using a TOF3D pulse sequence is developed and accelerated reliability growth test is performed. Section 4 presents the test results and failures followed by a Crow AMSAA plot. Finally, Section 5 concludes the paper.

## 2. Related work

### 2.1. Magnetic Resonance Imaging (MRI) System Brief Overview

The MRI system mainly consists of magnet, gradient, and radio frequency (RF) as critical subsystems. The magnet subsystem produces the main magnetic field. This magnetic field is applied in all three directions (X, Y, and Z). Afterwards, a magnetic gradient is applied in each axis, using gradient coil. Thus, magnetic field varies linearly along each axis. Gradient magnetic field is added or subtracted to the main magnetic field based on the applied gradient field. Due to varying magnetic field, resonance frequency is different for the protons at different places in the anatomy (human body), planned for imaging. The RF coil excites these protons by applying transmit power. Once transmit power is removed then protons relax, and it produces the reflected power. The reflected power is detected by same RF coil and amplified further. Reflected RF power forms the image dataset in the k-space. Fourier transform of the k-space produces the anatomical MRI image. There are various methods to apply magnetic gradient and RF power as per pulse sequence techniques. MRI pulse sequence plays an important role for MRI image based on patient body part and target diagnosis.

The MRI system is a very complex, expensive, and reparable product [1]. Hence a long life of 10 years is expected to maximize the value for the money to the customer [1]. During the long-life usage, the MRI system experiences many different failure modes and breakdowns. Some of these failures are quenching and overheating of the magnet, breaking and overheating of the gradient coil, breaking of the RF coil, and several other defects related to hardware and software failures [1]. During these failures in hospital, service engineers have to repair the system. Frequent failures of the MRI system, inside the hospital, reduce the system’s availability for a patient scan thus service costs are increased while quality is decreased. All these failures lead to an unhappy customer and higher cost of ownership. To improve the MRI product reliability, top MRI companies in the world perform parts and software reliability rigorously. However, it still has high defect rate and frequent service (almost once a month). Individual parts, subsystems or software reliability cannot catch the failure due to complex interaction between parts and parts, subsystems and subsystems, parts and software, subsystems and software, and software and software for a complex product like a MRI. To detect the hidden and unknown failures due to these complex interactions a system reliability test is desirable for a MRI product.

### 2.2. Types of Reliability Test

In last several decades, many reliability tests are developed and successfully implemented in different products to achieve high quality. Some of the system reliability tests are explained below:

#### 2.2.1. Reliability Growth Test

A reliability growth test is a method to perform reliability test to identify failures, fix these failures and continue the test (not needing to restart the test from beginning) after a failure, as introduced by J. T. Duane in 1964 [7]. It is a test for design growth of a new product especially during the development stage [4]. During new product development, design is weak and hence failures are expected. Hence, a reliability growth test gives flexibilities to continue the test after fixing the failure instead of restarting from the beginning. This is biggest advantage of growth test. Due to this flexibility, reliability growth test is adopted by many industries, including aircraft [7], defense [16], automotive [17], cellular telephone [18], solar [3] etc. This test can be applied to system, subsystem, parts, or software [6]. Another advantage of a reliability growth test is small sample size by the Bayes approach [8]. Hall and Mesh [19] introduce a framework for the evaluation of reliability growth with one sample size. Smaller sample size is very important for a complex and expensive MRI system.

#### 2.2.2. Reliability Growth Test Using Crow AMSAA Model

Larry H. Crow introduces the reliability analysis for complex and repairable systems in 1975 [8]. Crow AMSAA model is a relation between failures intensity (λ) over the time (t) as shown in Equation (1) [8,9,11]. The failure intensity (λ) depends on the shape parameter (β) and scale parameter (α) [8,9,11]. The shape and scale parameters are calculated based on rank regression [9], maximum likelihood estimation (MLE) [8,9] or the International Electrotechnical Commission (IEC) method [9]. For a repairable system reliability test, MLE fits best [8]. In the MLE method, reliability test can be terminated based on number of failures or test time [9]. We are going to use the MLE time-terminated approach for MRI system reliability test. For the MLE time-terminated case, shape and scale parameters are calculated in Equations (2) and (3), respectively [8,9]. Both shape and scale parameters are calculated based on number of failure (*N*), total test time (*T_s_*) and time at each failure (*T_i_*) occurring.

(1) $$\lambda =\alpha \times \;\beta \;\times \;t^{\beta -1}$$

(2) $$\beta =\frac{N}{\sum_{i=1}^{N}\ln\left(\frac{T_{s}}{T_{i}}\right)}$$

(3) $$\alpha =\frac{N}{T_{s}^{\beta }}$$

Here,

*λ* = Failure intensity
*α* = Scale parameter
*β* = Shape parameter
*N* = Total number of failures
*t* = Test time
*T_s_* = Total test time
*T_i_* = Time at ith failure occurring

**Figure 1:**
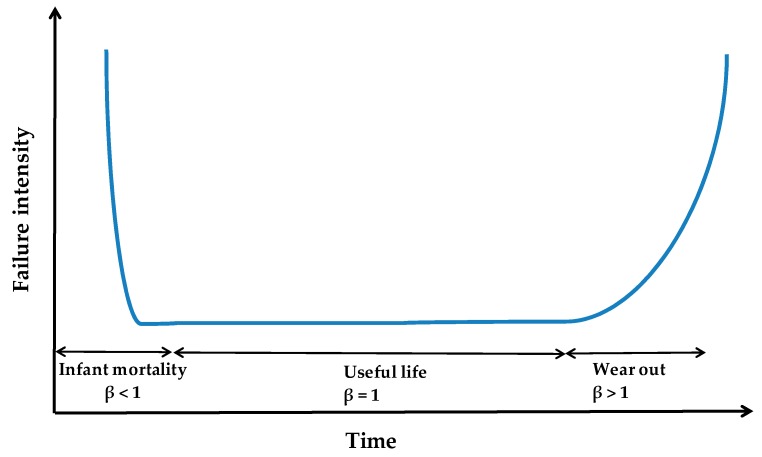
Bath-tub curve.

If we plot the failure intensity (λ) over the time *t*, it produces bath-tub curve as shown in Figure 1 [11]. Bath tub curve is divided into 3 sections [11,13]. First section is for β < 1, in which failures are consistently decreasing over the time [10,11,13]. This section is called as infant mortality or early life period [11]. During infant mortality, a new system encounters several unknown or hidden failures, which are not discovered before the product release. Second section is for β = 1, in which failures are almost constant [10,11]. This section is called as useful life period [11,13], which causes system failures due to unreliability of the product. The third section is for β > 1, where failures keep on increasing due to the system wearing out [10,11,13]. This means the system has completed its useful life.

During complex product development of a MRI system, if we perform the reliability growth test before product release then infant mortality failures are discovered proactively. Discovering these failures proactively before product launch improves the quality of the product. A reliability growth test also helps in understanding unreliability left in the product before launch. It helps to determine that product quality is at an acceptable level or not.

#### 2.2.3. Reliability Demonstration Test

A reliability demonstration test is performed to demonstrate that product meets the quality goal targeted during product planning phase [2]. It is performed during pilot production stage, which is after the verification stage and before mass production [2]. Usually, reliability demonstration is a zero-failure based test [2]. During a reliability demonstration test, failure is not allowed [20,21,22]. If failure occurs during the test, a demonstration test needs to restart from the beginning. This can lead to longer test duration and sample size issue.

#### 2.2.4. Accelerated Life Test

Usually, a life test is a common test method to prove the life of a part or non-repairable product. In this test, first of all we identify the stress parameters [23,24,25]. Once stress parameters are identified then we develop the test condition by elevating the stress condition to a level above the normal operating condition but below the design limit [23,24]. Later, we perform the test under this elevated stress condition [23,24,25]. As the normal operating condition of a part or product is much lower than the elevated stress condition, it gives acceleration for product aging. Accelerating the test gives the advantage of reducing the test time to respectively acceptable limit. If *AF*_1_, *AF*_2_, and *AF*_n_ are acceleration factors due to stress parameter 1, 2, and *n* then total acceleration factor is a multiple of acceleration factor due to individual stress as shown in Equation (4) [23,24,25]. Based on sample size, product life and total acceleration factor, test time is calculated by Equation (5).

(4) $$AF=AF_{1}\;\times \;AF_{2}\;\times \ldots \ldots .\;\times \;AF_{n}$$

(5) $$T=\;\frac{L}{AF\;\times \;s}$$

Here,

*AF*_1_ = Acceleration factor due to stress parameter 1
*AF*_2_ = Acceleration factor due to stress parameter 2
*AF_n_* = Acceleration factor due to stress parameter *n*
*AF* = Total acceleration factor
*L* = Product life
*s* = Sample size
*T* = Test time

An accelerated life test is widely used for electrical circuit board and parts [24]. Usually stress conditions are defined as higher temperature, humidity or other electrical parameters (voltage, current, power) [23,24]. Every stress parameter degrades the parts differently and hence degradation models are different [25]. Some of the degradation models are based on the Arrhenius, inverse power, Coffin–Manson, and Eyring concepts etc, which are widely known but not covered in this paper.

## 3. Proposed MRI System Accelerated Reliability Growth Test

### 3.1. Development of Nominal Day Usage Scenario for a MRI System

To perform system reliability test of MRI system, it is essential to analyze the actual hospital usage scenario, and workflow to correctly perform the reliability test. To do this, we collected the following data from different sources.

- Hospital 1: 50,867 exams on 8 MRI systems in a year
- Hospital 2: 53,099 exams on 8 MRI systems in a year
- NHS, England (Multiple Hospitals): 1,980,000 exams on 304 systems [26]

**Figure 2:**
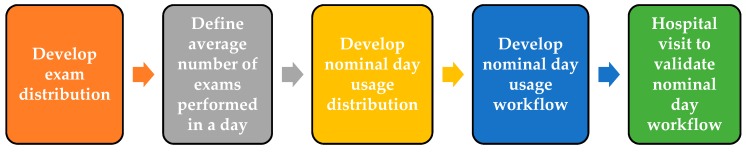
Strategy to develop ‘nominal day usage’ of a magnetic resonance imaging (MRI) system.

Hospital 1 and 2 are busy hospitals in the Republic of Korea and United States. Both hospitals have 8 MRI systems. Hospitals 1 and 2 have performed 50,867 and 53,099 exams in one year, respectively. We also collected MRI exams data from National Health Service (NHS), England. As per the NHS, approximately 1.98 million MRI exams are performed in one year by approximately 304 MRI systems. Based on these hospitals’ data, we developed a MRI system nominal day usages workflow strategy as shown in Figure 2.

#### 3.1.1. MRI Exam Distribution

We collected yearly data of MRI exams performed in two different hospitals. Based on collected data, we develop exam distribution. In hospital 1, approximately 50,867 exams and hospital 2 approximately 53,099 exams are performed in one year. These exams include brain, head/neck, spine (cervical or lumbar), extremities (hand, wrist, knee, ankle, shoulder and thigh), MR angiography, abdomen and other body parts as shown in Table 1. After data mining, exam distribution is developed based on the data of hospitals 1 and 2. Then, the exam distribution is further normalized with data obtained from other web sources. These web sources include the European Magnetic Resonance Forum (EMRF) [27] and Diagnostic Imaging Dataset (DID) of NHS England [26]. The normalization and data mining from different sources make MRI exam distribution very realistic as shown in Table 1. Our next step is to find out the average number of MRI exams performed in a day.

#### 3.1.2. Average Number of MRI Exams in a Day

Typically, MRI systems are used 6 days in a week and 50 weeks in a year in most of the hospital. Below is the analysis to determine average number of exams performed in a nominal day at different hospitals.

Number of exams per day per system in Hospital 1 = $\frac{50867}{50\;\times \;6\;\times \;8}=21.2$
Number of exams per day per system in Hospital 2 = $\frac{53099}{50\;\times \;6\;\times \;8}=22.1$
Number of exams per day per system as per NHS data = $\frac{1980000}{50\;\times \;6\;\times \;304}=21.7$
Average number of exams per day = $\frac{21.2+22.1+21.7}{3}=21.6$

Hospital 1 and 2 performed approximately 21.2 and 22.1 exams per day on a MRI system. As per NHS, England, on average 21.7 exams are performed on 304 systems. Further averaging of these data gives the average number of exams perform in a day for our project. In our work, we consider 21 exams are performed in a nominal day on a MRI system.

#### 3.1.3. Nominal Day Usage Distribution

We develop nominal day usage distribution as shown in Table 2 as per MRI exam distribution in Table 1 and average number of exams performed in a day as derived in Section 3.1.2. A typical target diagnosis is also defined with each exam type to determine correct pulse sequence technique to make it more realistic. Table 2 consists of 21 different exams, which are divided into 10 brain exams, 1 head and neck exam, 3 spine exams, 2 extremity exams, 3 abdomen exams, and 2 angiography exams.

#### 3.1.4. Nominal Day Usage Workflow

Table 3 shows the workflow of nominal day usage, which is developed based on nominal day exam distribution in Table 2. Table 3 consists of exam number (#), exam type, target diagnosis, contrast used, RF coil type, and description of each exam step. The description/scan protocol column also defines the pulse sequences used in each exam.

We determine the type of RF coil and contrast needed for each exam as per target diagnosis. We have several RF coils named as head, neck, spine, or extremities RF coils. Some exams use contrast based on target diagnosis. All these variations are listed in Table 3 to make usage scenario more realistic before conducting a reliability test. Table 3 has approximately 393 rows, which are not shown in this paper. We listed all steps for exam 1 and few steps of exam 2 and 21 to give an understanding of nominal day usage workflow developed for a MRI system.

#### 3.1.5. Hospital Visit to Validate the Workflow

In the last step of developing the nominal day usage scenario, we went to four different hospitals and validated the daily usage workflow. During validation, we found brain exams are performed more than spine exams. Hence, a small adjustment is undertaken by increasing one brain exam and reducing a spine exam in Table 2 and Table 3.

### 3.2. MRI System Reliability Growth Test and Current Challenges

In order to perform a reliability growth test based on nominal day usage as developed in Table 3, we need to find out test time to complete 21 exams in a day. After careful study, we understand that out of all steps in Table 3, pulse sequence steps stress the MRI system extensively. These pulse sequence steps for exam 1 are the localizer, T1 spin echo transverse (T1 SE TRA), T2 fluid-attenuated inversion recovery transverse (T2 Flair TRA), T2 turbo spin echo transverse (T2 TSE TRA), diffusion-weighted imaging (DWI), T2* fast low-angle shot 2-dimensional transverse (T2* FL2D TRA). Based on these understandings, Table III is reconstructed considering pulse sequences (PS) steps as shown in Table 4. We added time required to complete each pulse sequence in Table 4 column entitled “PS Time”. This gives one nominal day test time as 15,387 s or 4.27 h.

We assume that the test is performed 24 h per day and 7 days per week, then time to complete the reliability test can be calculated by Equation (6) as below.

(6) $$T=\;\frac{L\;\times \;W\;\times \;D\;\times \;TD}{H}$$

(7) $$\mathrm{TD}\;=\sum_{i=1,\;j=1}^{i=n,\;j=m}T_{ij}=\;T_{11}+\;T_{12}+\ldots +\;T_{1n}+\ldots +\;T_{m1}+\;T_{m2}+\ldots +\;T_{mn}$$

Here,

*L* = MRI system life in years
*W* = Number of weeks per year for MRI system usage
*D* = Number of nominal days per week MRI System usage
*n* = Number of exams performed in a nominal day
*m* = Number of pulse sequence in each exam
*i* = *i*th exam performed in a nominal day
*j* = *j*th pulse sequence
*PSij* = *j*th pulse sequence of *i*th exam
*Tij* = Time taken by *j*th pulse sequence in *i*th exam
*TD* = Time to complete all exams in a nominal day
*T* = Time (in days) to complete the reliability growth test
*H* = Number of test hours in a day

As per Equation (7), TD is calculated in Table 4 in PS Time column.

TD = 15,387 s = 4.27 h.

Usually, MRI system service life is at least 10 years [1]. As per Section 3.1.2 of this paper, MRI system yearly usages are defined as 50 weeks in a year and 6 days per week. Average number of exams perform in a day is 21.

*L* = 10 years
*W* = 50 weeks/year
*D* = 6 days/week
*n* = 21
*H* = 24 h

From Equation (6), Test Time (T) = 537 days.

We consider 537 days a very long test duration to perform a system reliability test during the product development phase. Most of the MRI product manufacturers cannot afford 537 days to undertake a long reliability test due to limitations like the pressure of the product launch, cost etc.

### 3.3. MRI System Stress Parameters and Life-Time Stress Analysis

To accelerate the MRI system reliability test and reduce the test duration, it is essential to identify the system stress parameters. Using these stress parameters, we need to calculate the life-time stress for a MRI system using nominal day usage scenario.

#### 3.3.1. Identifying Stress Parameters

As discussed in Section 2.1, MRI system undergoes through various kinds of stress every day. Based on our analysis, we found that some of these stresses are; magnet pressure, cold head temperature, gradient coil temperature, gradient coil vibration, RF coil applied power, RF power, gradient power, and input current etc. These stresses are at peak, while pulse sequence is applied as explained in Section 3.2. To reduce the system reliability test duration (537 days as calculated in Section 3.2), we need to accelerate the test. We found vibration energy of the gradient coil as the most suitable stress parameter, which gives the highest acceleration factor to accelerate the reliability test.

#### 3.3.2. Establishing Relation Between Pulse Sequences and Vibration Energy

**Figure 3:**
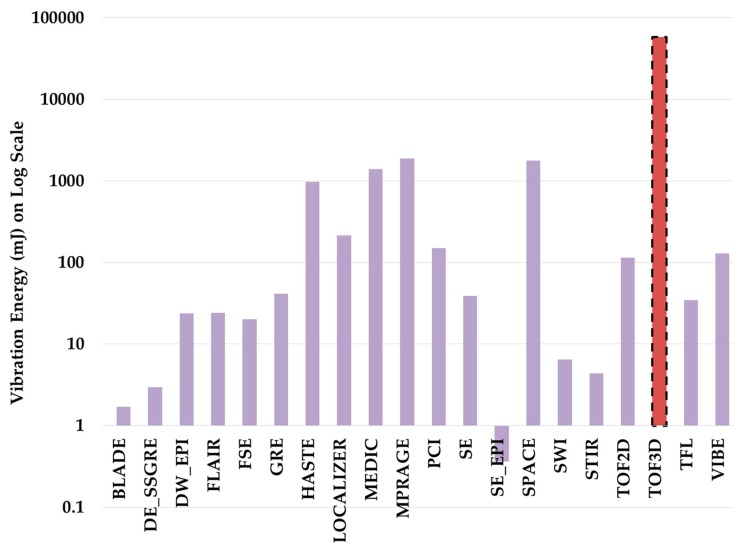
Sequence vs. vibration energy of MRI gradient coil.

We develop a simulation model to calculate vibration energy exerted on a gradient coil by different pulse sequence parameters. Figure 3 shows vibration energy is applied on gradient coil by different pulse sequences. As demonstrated in Figure 3, the TOF3D pulse sequence (highlighted) exerts maximum vibration energy of 58J to the gradient coil as compared to all other pulse sequence techniques. Hence, we constructed a reliability growth test cycle using TOF3D pulse sequence to get a high acceleration factor.

#### 3.3.3. Life-Time Analysis using Vibration Energy

Table 4 depicts the nominal day usage profile, which consists of 21 exams. Each exam has predefined pulse sequences based on target diagnosis of anatomy. Gradient coil vibration energy is calculated for each pulse sequences of all exams in the last column of Table 4 tilted as “vibration energy”. The total vibration energy in a day is calculated in Equation (8), which is the sum of all energy in the last column of Table 4. Based on one nominal day’s vibration energy, life-time vibration energy is calculated in Equation (9).

(8) $$\begin{array}{ll} VE_{D}\; & =\sum_{i=1,j=1}^{i=n,j=m}VE_{ij}\\ & \;=\;VE_{11}+\;VE_{12}+\ldots +\;VE_{1n}+\ldots +\;VE_{m1}+\;VE_{m2}+\ldots +\;VE_{mn} \end{array}$$

(9) $$VE_{T}\;=L\;\times \;W\;\times \;D\;\times \;VE_{D}$$

Here,

*VE_ij_* = Vibration energy exerted on gradient coil during *j*th pulse sequence in *i*th exam
*VE_D_* = Total vibration energy exerted on gradient coil in a nominal day
*VE_T_* = Total vibration energy exerted on gradient coil in entire life

### 3.4. MRI System Accelerated Reliability Growth Test

We need to develop a reliability growth test cycle, which can stress the system as much possible but within the system design limit. 

#### 3.4.1. Developing Test Cycle to Accelerate the Reliability Test

As discussed in Section 3.3.2, vibration energy applied on the MRI system is highest during TOF3D pulse sequence. Based on many permutations and combinations, a test cycle is developed consisting of 10 TOF3D pulse sequences and idle time as shown in Table 5. The first column of Table 5 defines the steps between TOF3D and idle time. Each TOF3D pulse sequence takes approximately 410 s to complete and exerts 58.03 joules of vibration energy on a gradient coil as shown in second and third column of Table 5. Cumulative energy is total energy consumed by gradient until the ongoing step as shown in last column of Table 5. An idle time of 60 s is planned between two TOF3D pulse sequences. It is obvious that vibration energy during idle duration is zero. It takes approximately 8240 s or 2.29 h to complete a test cycle. During a test cycle, the gradient coil undergoes through approximately 580.3 joules of vibration energy. To prevent the magnet quench, each test cycle has one hour break time for system to cool down as added in the last row of Table 5.

#### 3.4.2. Calculating Acceleration Factor and Test Duration

As we calculate the vibration energy for the life time and a test cycle from Section 3.3.3 and Section 3.4.1, we estimate the acceleration factor (*AF_v_*) in Equation (10). We consider the inverse power law model of vibration energy to calculate the acceleration factor in Equation (10) [25]. Here p is the inverse power law coefficient [25]. Similarly, we can also calculate the acceleration factor due to time (*AF_T_*) in Equation (11) using hours of test in a day (H) and time to complete one test cycle (*T_c_*). The daily acceleration factor for the test (*AF*) can be calculated in Equation (12) by multiplying acceleration factors due to vibration energy and time. Time to complete system reliability growth test (*T*) is calculated in Equation (13). Total test time (*T*) is the division of life in days divided by multiple of total acceleration factor (AF) and sample size (s). If we put *VE_D_* from Equation (8) in Equation (10) then test time is calculated by Equation (14).

(10) $$AF_{V}={\left(\frac{VE_{C}}{VE_{D}}\right)}^{p}$$

(11) $$AF_{T}=\frac{H}{T_{c}}$$

(12) $$AF=AF_{V}\;\times \;AF_{T}$$

(13) $$T=\frac{L\;\times \;W\;\times \;D}{AF\;\times \;s}$$

(14) $$T=\frac{L\;\times \;W\;\times \;D\;\times \;{\left(\sum_{i=1,j=1}^{i=n,j=m}VE_{ij}\right)}^{p}\;\times \;T_{c}}{VE_{C}^{p}\;\times \;H\;\times \;s}$$

Here,

*VE_c_* = Total vibration energy in a test cycle
*VE_D_* = Total vibration energy exerted on gradient coil in a nominal day
*p* = Inverse square law coefficient for vibration
*T_c_* = Time to complete one test cycle
*H* = Number of test hours in a day
*T* = Time (in days) to complete the reliability growth test
*AF_V_* = Acceleration factor due to vibration energy
*AF_T_* = Acceleration factor due to time
*AF* = Total acceleration factor
*s* = Sample size (Number of test sample)
*L* = MRI system life in years
*W* = Number of weeks per year for MRI system usage
*D* = Number of nominal days per week MRI System usage
*n* = Number of exams performed in a nominal day
*m* = Number of pulse sequence in each exam
*i* = *i*th exam performed in a nominal day
*j* = *j*th pulse sequence
*PS_ij_* = *j*th pulse sequence of ith exam
*VE_ij_* = Vibration energy exerted on gradient coil during *j*th pulse sequence in ith exam

Following parameters are calculated for our accelerated growth test on a target MRI system:

*VE_c_* = 580.3 Joules (from Table 5)
*VE_D_* = 193.3 Joules (from Table 4)
*p* = 1.5 [25]
*T_c_* = 8240 s = 2.29 h (from Table 5)
*H* = 24 h/day
*s* = 1 (sample size as one for expensive system and long-life MRI product [2])
*L* = 10 years [1]
*W* = 50 weeks/year
*D* = 6 days/week

From Equations (10), (11), (12), and (13), test duration can be calculated as follows. Accelerated system reliability growth test duration is reduced from 537 days to 55 days, which is a remarkable achievement for a MRI system with one sample size.

$$AF_{V}={\left(\frac{580.3}{193.3}\right)}^{1.5}=5.2$$

$$AF_{T}=\frac{24}{2.29}=\;10.48$$

AF=5.2 × 10.48=54.5

$$T=\frac{10\;\times \;50\;\times \;6}{54.5\;\times \;1}=55\;days$$

#### 3.4.3. Performing an Accelerated Reliability Growth Test

Our growth test is performed as per the TOF3D-based pulse sequence test cycle developed in Section 3.4.1. We observed the system break down several times due to the magnet, gradient, RF, and software subsystems during the initial phase of the reliability growth test. These failures are fixed and the test is continued until it achieved system design maturity. The system has log capability to monitor many parameters to check performance of the system during the reliability growth test. Some of these parameters are listed below:

- Magnet pressure;
- Magnet body temperature;
- Gradient coil temperature;
- Heat exchanger unit coolant temperature;
- RF amplifier coolant temperature;
- Gradient amplifier coolant temperature;
- Gradient coil coolant temperature;
- Several other parameters for software and system.

## 4. Accelerated Reliability Growth Test Result and Discussion

The MRI system reliability growth test is performed for more than 55 days. Several parameters are logged. These parameters are analyzed every day to check for degradation or failure. During the test, both soft and hard failures have happened. Soft failures are those failures that are self-recoverable without any software or hardware modification after restarting or rebooting the subsystem or system [25]. Hard failures are those failures that are not self-recoverable and need software or hardware modifications to restart the test [25]. We observed 12 hard failures during the reliability growth test. Some of these failures are described in subsequent sections followed by the Crow AMSAA plot.

### 4.1. Magnet Subsystem Performance

**Figure 4:**
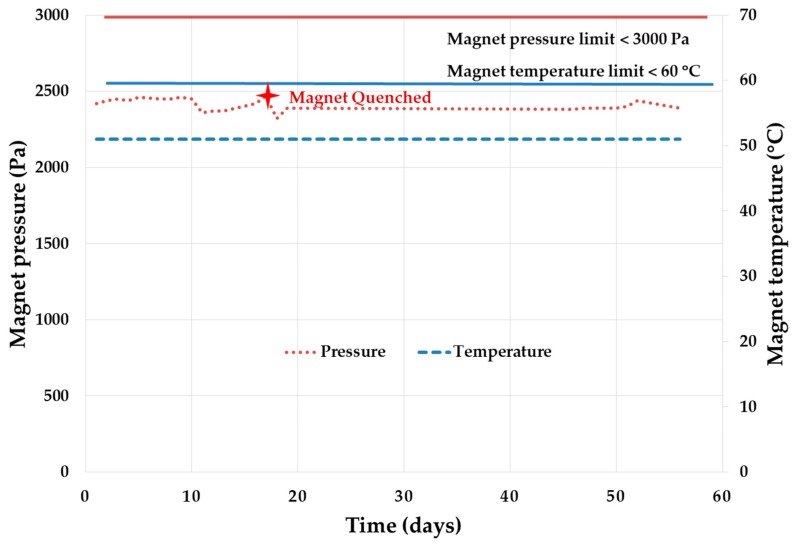
Magnet subsystem performance during MRI system reliability growth test.

The magnet pressure and magnet body temperature over the period is illustrated in Figure 4. On the 17th test day, the magnet has quenched even though magnet pressure and temperature are within the range. The main root cause of the quench is coolant impurity due to repeated exams as the adsorber reached the end of its life. After replacing the adsorber (coolant filter), the test restarted and continued. This helps to determine the adsorber as a serviceable part with predefined planned maintenance every year.

### 4.2. Gradient Subsystem Performance

**Figure 5:**
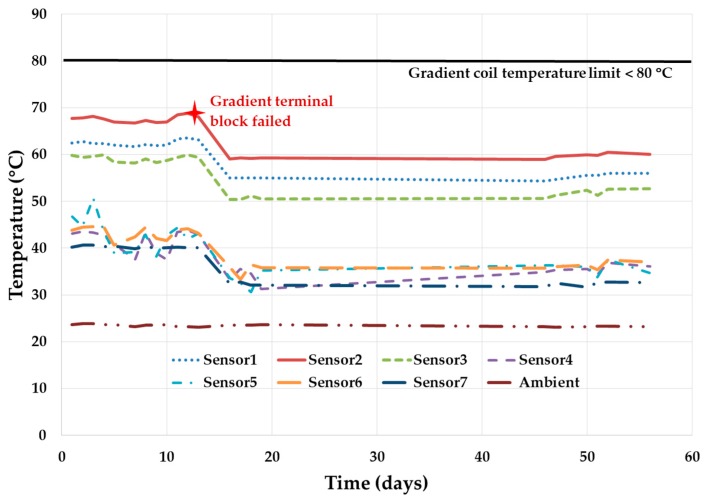
Gradient subsystem performance during MRI system reliability growth test.

Figure 5 shows the gradient coil temperature at seven different locations. Even though temperature is within the specified limits, the gradient coil terminal block caught fire on the thirteenth day of test. Terminal block is designed with suitable material and clearance between the phases in order to prevent this kind of failure. However, this hidden failure still occurred, which was discovered during the reliability growth test. The terminal block was redesigned and replaced, and the reliability growth test continued again. This catastrophic hidden failure was discovered and fixed proactively before it could happen at the customer’s location. Additionally, the heat exchanger unit (HEU) coolant flow rate was increased to extract the gradient coil heat out more efficiently. This reduced the temperature of the gradient coil and all its sensors’ reading for a stable performance as shown in Figure 5.

### 4.3. Crow AMSAA Plot

During the MRI system reliability growth test, approximately 12 hard failures (*N*) were found in 60 days of the test. Hence, total test time (*T_s_*) was approximately 60 days or 1440 h. All failures were recorded with respect to failure time (*T_i_*). Based on these values (*T_s_* and *T_i_*), the shape parameter (β) was calculated as 0.304 as per Equation (2). The scale parameter (α) was calculated as 1.362 as per Equation (3). Putting the value α and β of in Equation (1) [8,9], the Crow AMSAA model for MRI system reliability test is presented in Equation (15).

*N* = 12
T_s_ = 1440 h
*T_i_* = 2, 24, ….., 1080 h

From Equation (2) shape parameter was calculated,

β=0.304

From Equation (3), scale parameter was calculated:

$$\alpha =\frac{12}{1440^{0.304}}=1.362$$

From Equation (1), failure intensity was calculated:

$$\lambda =1.362\;\times \;0.304\;\times \;t^{0.304-1}$$

(15) $$\lambda =0.415\;\times \;t^{-0.696}$$

**Figure 6:**
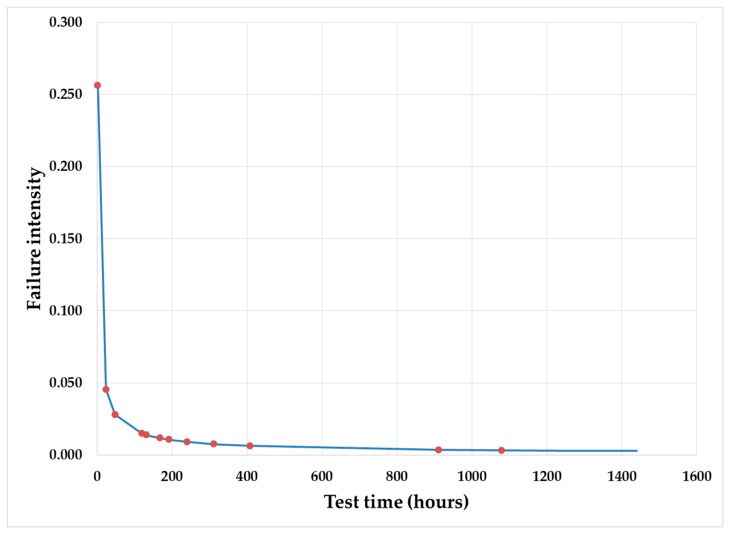
Crow AMSAA plot for MRI system reliability growth test.

From Equation (15), the Crow AMSAA plot was developed for MRI system reliability growth test. The plot is shown in Figure 6. From the Crow AMSAA plot, it is very clear that the MRI system has crossed the infant mortality phase (failure intensity decreasing rapidly) and reaches its useful life period (failure intensity is almost constant) as explained in Section 2.2.2. This proves that the MRI system has reached system design maturity.

## 5. Conclusions

We presented a novel idea to perform a MRI system reliability growth test in an acceptable test time of 55 days. The test was performed during the product development phase on one sample size. We constructed a test cycle consisting of a high-energy TOF3D pulse sequence. This method accelerated the reliability growth test and reduced the test duration from 537 to 55 days. The accelerated reliability growth test was successfully performed on a MRI system for 60 days using the TOF3D test cycle representing a hospital usage scenario. Many hidden failures were discovered and fixed during the test. We successfully implemented the Crow AMSAA model and demonstrated that the MRI system reliability was in a useful life not an infant mortality phase. This concept of performing an accelerated system reliability growth test based on a field usage scenario can also be applied in other complex, long-life, and high-cost products.

## Figures and Tables

**Table 1:** MRI exam distribution (%).

Exam Type	Hospital 1	Hospital 2	EMRF [27]	NHS [26]	Normalized Distribution
Brain	50	43	25	29	32
Head/Neck	6	1	6	9	7
Spine	15	15	25	36	19
Extremities	7	6	20		11
MR Angio	0	15	9	0	8
Abdomen	19	10	8	15	14
Other	4	9	7	11	9

**Table 2:** Nominal day exam distribution.

Exam Number (#)	Exam Type	Target Diagnosis
1	Brain	Transient ischemic attack
2	Brain	Demyelinating
3	Brain	Routine
4	Brain	Routine with contrast
5	Brain	Brain tumor
6	Brain	Transient ischemic attack
7	Brain	Demyelinating
8	Brain	Routine
9	Brain	Routine with contrast
10	Brain	Brain tumor
11	Head and neck	Head and neck routing
12	Spine	Cervical basic
13	Spine	Thoracic spin basic
14	Spine	Lumber trauma
15	Extremities	Knee meniscus
16	Extremities	Shoulder
17	Abdomen and liver	General abdomen/pelvis
18	Abdomen and liver	Liver routine
19	Abdomen and liver	Liver steatosis/fibrosis
20	Angiography	Brain angiography
21	Angiography	Whole body angiography

**Table 3:** Nominal day usage workflow of MRI system.

#	Exam Type	Target Diagnosis	Contrast	Coil Type	Description/Scan Protocol
1	Brain	Transient Ischemic	No	Head	Patient go inside scan room
1	Brain	Transient Ischemic	No	Head	Patient lie down, RF coil setup
1	Brain	Transient Ischemic	No	Head	Table moved up and slide in
1	Brain	Transient Ischemic	No	Head	Turn on laser, Patient landmark
1	Brain	Transient Ischemic	No	Head	Table moved to home position
1	Brain	Transient Ischemic	No	Head	Localizer
1	Brain	Transient Ischemic	No	Head	Set the FOV and any parameters
1	Brain	Transient Ischemic	No	Head	T1 SE TRA
1	Brain	Transient Ischemic	No	Head	T2 Flair TRA
1	Brain	Transient Ischemic	No	Head	T2 TSE TRA
1	Brain	Transient Ischemic	No	Head	DWI
1	Brain	Transient Ischemic	No	Head	T2* FL2D TRA
1	Brain	Transient Ischemic	No	Head	Image review and saved to PACS
1	Brain	Transient Ischemic	No	Head	Table slide out and lowered down
1	Brain	Transient Ischemic	No	Head	RF coil removed
1	Brain	Transient Ischemic	No	Head	Patient moved from scan room
1	Brain	Transient Ischemic	No	Head	Break time
2	Brain	Demyelinating	No	Head	Patient go inside scan room
2	Brain	Demyelinating	No	Head	Patient lie down, RF coil setup
2	Brain	Demyelinating	No	Head	Table moved up and slide in
2	Brain	Demyelinating	No	Head	Turn on laser, Patient landmark
2	Brain	Demyelinating	No	Head	Table moved to home position
2	Brain	Demyelinating	No	Head	Localizer
.	.	.	.	.	.
.	.	.	.	.	.
.	.	.	.	.	.
21	MR Angio	Whole Body Angio	No	Multiple	FL3D VIBE @ Top
21	MR Angio	Whole Body Angio	No	Multiple	FL3D COR PRE POST @ Top
21	MR Angio	Whole Body Angio	No	Multiple	Image review & saved to PACS
21	MR Angio	Whole Body Angio	No	Multiple	Table slide out & lowered down
21	MR Angio	Whole Body Angio	No	Multiple	RF coil removed
21	MR Angio	Whole Body Angio	No	Multiple	Patient moved from scan room

T2* FL2D TRA: T2* fast low-angle shot 2-dimensional transverse.

**Table 4:** Nominal day test time calculation.

#	Exam Type	Target Diagnosis	Pulse Sequence	Pulse Sequence Time (s)	Vibration Energy (Joule)
1	Brain	Transient Ischemic	Localizer	12.4	0.214
1	Brain	Transient Ischemic	T1 SE TRA	76.9	0.039
1	Brain	Transient Ischemic	T2 Flair TRA	145.5	0.024
1	Brain	Transient Ischemic	T2 TSE TRA	17.5	0.02
1	Brain	Transient Ischemic	DWI	161.5	0.024
1	Brain	Transient Ischemic	T2*_FL2D_TRA	4.1	0.041
1	Brain	Transient Ischemic	Break time	306	0
2	Brain	Demyelinating	Localizer	12.4	0.214
2	Brain	Demyelinating	T1 SE TRA	76.9	0.039
2	Brain	Demyelinating	T2 Flair TRA	145.5	0.024
2	Brain	Demyelinating	T2 Flair SAG	145.5	0.024
2	Brain	Demyelinating	T2 TSE TRA	17.5	0.02
2	Brain	Demyelinating	T1 SE TRA	76.9	0.039
2	Brain	Demyelinating	Break time	306	0
.	.	.	.	.	.
.	.	.	.	.	.
21	MR Angio	Whole Body Angio	Localizer @ Bottom	12.4	0.214
21	MR Angio	Whole Body Angio	Localizer @ Middle	12.4	0.214
21	MR Angio	Whole Body Angio	Localizer @ Top	12.4	0.214
21	MR Angio	Whole Body Angio	FL3D COR @ Bottom	4.1	0.041
21	MR Angio	Whole Body Angio	FL3D COR @ Middle	4.1	0.041
21	MR Angio	Whole Body Angio	FL3D COR @ Top	4.1	0.041
21	MR Angio	Whole Body Angio	FL3D VIBE @ Bottom	41.5	0.128
21	MR Angio	Whole Body Angio	FL3D COR @ Bottom	4.1	0.041
21	MR Angio	Whole Body Angio	FL3D VIBE @ Middle	41.5	0.128
21	MR Angio	Whole Body Angio	FL3D COR @ Middle	4.1	0.041
21	MR Angio	Whole Body Angio	FL3D VIBE @ Top	41.5	0.128
21	MR Angio	Whole Body Angio	FL3D COR @ Top	4.1	0.041
				15387 Sec	193.3 Joule
				TD	VE_D_

T2*_FL2D_TRA: T2* fast low-angle shot 2-dimensional transverse.

**Table 5:** Test cycle to perform accelerated reliability growth test.

Pulse Sequence (PS)	PS Time (s)	Vibration Energy (Joules)	Cumulative Vibration Energy (Joules)
TOF3D	410	58.03	58.03
Idle Time	60	0	58.03
TOF3D	410	58.03	116.059
Idle Time	60	0	116.059
TOF3D	410	58.03	174.089
Idle Time	60	0	174.089
TOF3D	410	58.03	232.118
Idle Time	60	0	232.118
TOF3D	410	58.03	290.148
Idle Time	60	0	290.148
TOF3D	410	58.03	348.177
Idle Time	60	0	348.177
TOF3D	410	58.03	406.207
Idle Time	60	0	406.207
TOF3D	410	58.03	464.236
Idle Time	60	0	464.236
TOF3D	410	58.03	522.266
Idle Time	60	0	522.266
TOF3D	410	58.03	580.295
Break	3600	0	580.295
	8240 s (*T_c_*)	580.3 Joules (*VE_c_*)	
